# Ultrasound-Assisted Enhancement of Bioactive Compounds in Amazonian Fruit Juices (*Mammea americana*, *Solanum Sessiliflorum*, and *Cassia leiandra*)

**DOI:** 10.1021/acsomega.5c08708

**Published:** 2026-02-12

**Authors:** Célio Matias Airone Macalia, Josiana Moreira Mar, Adriano de Souza Carolino, Ronald Zico de Aguiar Nunes, Kalil Araújo da Silva, Camila Macena Ruzo, Jaqueline de Araújo Bezerra, Samuel Oliveira da Silva, Alessandra Ramos Lima, Edgar Aparecido Sanches

**Affiliations:** † Laboratory of Nanostructured Polymers (NANOPOL), 67892 Federal University of Amazonas (UFAM), Manaus, AM 69067-005, Brazil; ‡ Graduate Program in Materials Science and Engineering (PPGCEM), Federal University of Amazonas (UFAM), Manaus, AM 69067-005, Brazil; § Center for Studies in Science and Technology (NECET), Department of Science, Engineering, Technology, and Mathematics, Higher Institute of Rural Development and Biosciences (ISDRB), Rovuma University, Lichinga, Niassa 544, Mozambique; ∥ Graduate Program in Biotechnology (PPGBIOTEC), Federal University of Amazonas (UFAM), Manaus, AM 69020-120, Brazil; ⊥ Analytical Center, Federal Institute of Education, Science and Technology of Amazonas (IFAM), Manaus, AM 69020-120, Brazil; # Graduate Program in Chemistry (PPGQ), 67892 Federal University of Amazonas (UFAM), Manaus, AM 69020-120, Brazil; ∇ Environmental Biophotonics Laboratory, São Carlos Institute of Physics (IFSC), University of São Paulo (USP), São Carlos, SP 13566-590, Brazil

## Abstract

Despite growing interest in nonthermal technologies, the effects of ultrasound processing on the molecular and bioactive properties of underexplored Amazonian fruits remain poorly understood. This study provides an integrated physicochemical, spectroscopic, and antioxidant assessment of Abricó (*Mammea americana*), Cubiu (*Solanum sessiliflorum* Dunal), and Mari-mari (*Cassia leiandra* Banth) juices subjected to ultrasound treatment (20–80% power level). Moderate sonication (60%) significantly enhanced carotenoid and phenolic extraction without affecting pH, titratable acidity, or soluble solids, confirming the gentle, nonthermal character of the process. UV–Vis and FTIR analyses revealed preserved molecular fingerprints and characteristic π–π* transitions of carotenoids, polyphenols, and flavonoids, demonstrating structural stability after sonication. Antioxidant assays (DPPH, ABTS, and FRAP) showed increased radical-scavenging activity, particularly in Mari-mari juice, supported by PCA and Pearson correlation analyses. Color parameters (L*, a*, b*) shifted notably at 40% potency in Abricó and Cubiu juices, reflecting subtle but beneficial compositional changes associated with enhanced β-carotene release. Abricó juice exhibited higher moisture (15.49%) and lower lipid contents. Bioactive release was most evident in Abricó and Cubiu juices, which showed elevated total phenolics (138.00 ± 1.03 mg GAE·mL^–1^) and strong DPPH activity (1204 ± 3.82 and 1125 ± 3.63 μmol TE·100 mL^–1^, respectively) at 60% power level. In contrast, Mari-mari juice displayed no significant response to ultrasound. Untreated samples also presented high ABTS activity (2028.55 ± 4.44 μM TE). Overall, 60% power level emerged as an effective, economical, and eco-friendly strategy to enhance phenolic and carotenoid release in the tested Amazonian fruit matrices.

## Graphical abstract

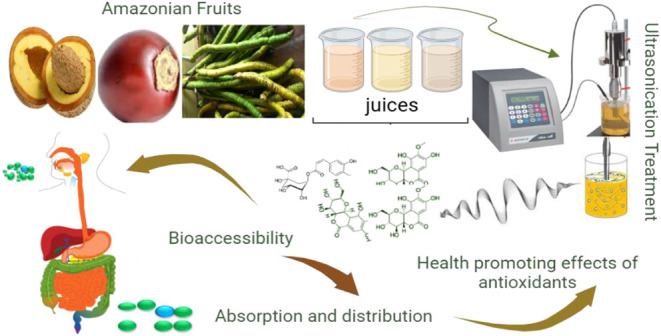

## 1 Introduction

The Amazon rainforest, one of the most biodiverse ecosystems on the planet, harbors an exceptional diversity of nutrient-rich fruits, seeds, and roots. Many of these native species are noteworthy sources of bioactive compounds such as carotenoids, anthocyanins, and phenolic constituents, which are recognized for their antioxidants, anti-inflammatory, and protective effects against chronic diseases.^1,2^ Among these species, *Mammea americana* L. (Abricó), *Solanum sessiliflorum* Dunal (Cubiu), and *Cassia leiandra* Benth (Mari-mari) stand out as underexplored Unconventional Food Plants (UFPs) with considerable nutritional and nutraceutical potential. However, their high fiber content and structurally complex matrices can hinder the release and intestinal absorption of bioactive compounds, reducing their overall bioavailability. Conventional extraction methods such as solvent extraction, distillation, and mechanical pressing—often show limited efficiency and may cause thermal or chemical degradation of sensitive metabolites due to elevated temperatures or the use of toxic solvents.^3–6^

Recent advances highlight a growing shift toward green and nonthermal extraction technologies, particularly ultrasound-assisted extraction (UAE). Through acoustic cavitation, UAE promotes cell wall disruption and enhances the release of lipophilic pigments and phenolic compounds, while reducing solvent consumption, energy requirements, and environmental impact.^5^ In this context, UAE has been widely explored as an alternative to conventional extraction techniques, mainly due to its ability to intensify mass transfer while preserving thermolabile compounds. Because many bioactive compounds remain trapped within plant cellular structures and are not fully released during digestion, UAE has also been proposed as an effective strategy to improve their bioavailability. In support of this, studies with puree,^7^ guava juice,^8^ and tomato juice^9^ report increased bioaccessibility of bioactive compounds following ultrasound processing.

Applications in Amazon fruits such as açaí, buriti, cubiu, camu–camu, and pomegranate further demonstrate the versatility and efficiency of UAE for recovering bioactive constituents from natural matrices.^4,10–13^ However, most previous studies have focused on high-energy ultrasound systems, the use of organic solvents, or conventional fruit matrices, with limited emphasis on water-based, moderate-intensity UAE conditions optimized for unconventional edible plants from the Amazon biome. Moreover, investigations combining UAE with *in vitro* digestion models to assess the bioaccessibility of bioactive compounds in Amazonian UFPs remain scarce.

Therefore, despite the growing body of literature on UAE, a clear research gap persists regarding the application of optimized, solvent-free extraction medium for wild Amazonian plant matrices, as well as its effects on the physicochemical, nutritional, and digestive stability of the extracted compounds. To address this scientific gap, the present study investigates a water-based, ultrasound-assisted approach to enhance both the extraction and the bioaccessibility of bioactive compounds of Abricó, Cubiu, and Mari-mari juices. This work contributes to the development of sustainable and high-value nutraceutical ingredients derived from Amazonian biodiversity by demonstrating the potential of UAE as an environmentally friendly and efficient processing technology.

## 2 Experimental Section

### 2.1 Sequential Steps in the Preparation of Juices

Bioactive compounds and dietary fibers were extracted and characterized from Abricó, Cubiu, and Mari-mari juices through a sequential workflow involving pulp processing and juice preparation, followed by physicochemical analyses, antioxidant assays, and spectroscopic characterization (UV–Vis, FTIR). Principal Component Analysis (PCA) was applied to identify compositional patterns among the samples, and *in vitro* digestibility was evaluated using simulated gastric and intestinal phases. Figure 1 summarizes the main steps involved in the extraction and release of bioactive compounds.

**Figure 1:**
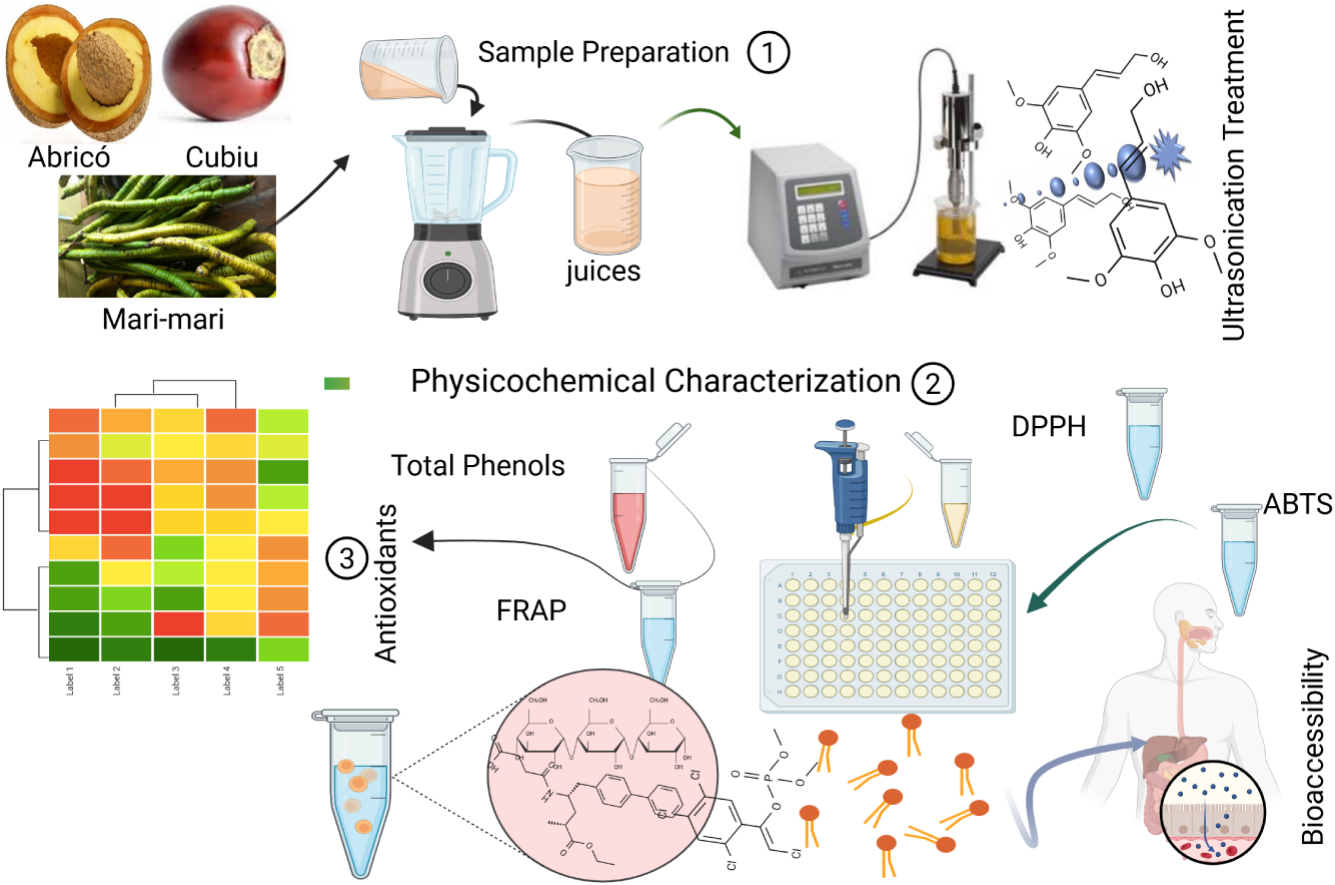
Workflow for juice preparation, physicochemical analysis, antioxidant assays, and chemical characterization.

### 2.2 Raw Material Acquisition and Preparation of Fruit Juices

Fruits *in natura* were purchased from the Producer’s Fair located at Av. Autaz Mirim, Cidade Nova, Manaus, AM, Brazil. After acquisition, the fruits were washed in potable water, followed by sanitization in a sodium hypochlorite solution for 10 min and subsequent rinsing under running tap water. The cleaned fruits were then peeled, weighed using an analytical balance, and processed in a blender (Oster Super BLSTMG-BR8). The resulting pulp was sieved, refrigerated at 10 °C, and subsequently lyophilized. Juices were prepared by reconstituting the lyophilized pulp at a 1:2 ratio (g/mL pulp-to-water) to a final volume of 200 mL. All analyses were conducted in triplicate. Figure 2 shows the fruits *in natura*.

**Figure 2:**
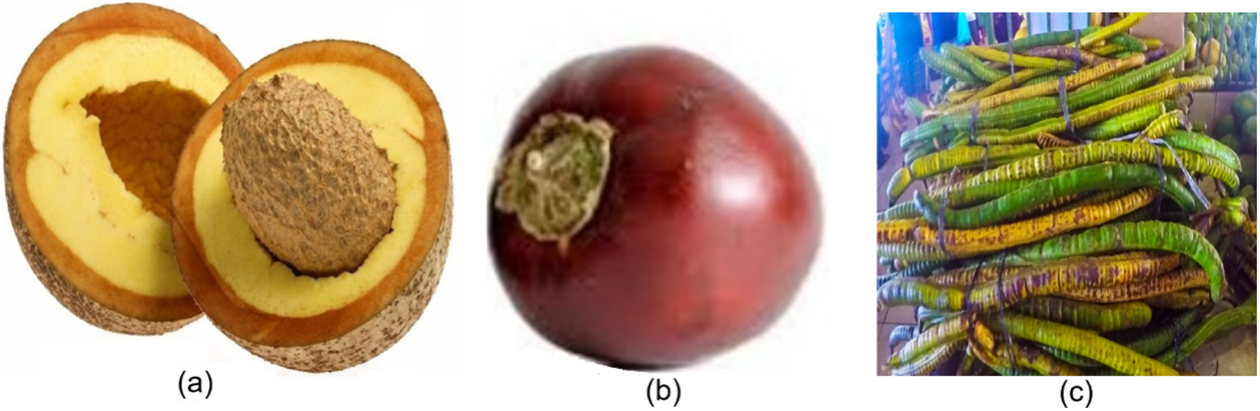
Photographs of the Amazonian fruit species used in this study: (a) *Mammea americana* L. (Abricó), (b) *Solanum sessiliflorum* Dunal (Cubiu), and (c) *Cassia leiandra* Benth (Mari-mari).

### 2.3 Juice Processing

The juices of each species were divided into five treatment groups: an untreated control and ultrasound treatments at 20% (150 W; US20), 40% (300 W; US40), 60% (450 W; US60), and 80% (600 W; US80). All samples were processed using ultrasonic homogenization for 10 min, with temperature maintained below 45 °C. Ultrasound was applied with a 25 mm probe operating at 20 kHz in a Vibra-Cell VCX 750 tip sonicator (Sonics & Materials, Inc., Newtown, CT, USA).

### 2.4 Color Parameters

Colorimetric parameters were measured using a digital colorimeter (Delta Color 71421, Delta Vista), which provided luminosity (L*), red-green coordinate (*a*), and yellow-blue coordinate (b*). All measurements were performed in triplicate. The total color difference (Δ*E*) was calculated according to eq 1.

1 $$\Delta E^{*}=\sqrt{\Delta L^{*2}+\Delta a^{*2}+\Delta b^{*2}}$$

### 2.5 Total Phenolic Content

The Total Phenolic Content (TPC) of fruit juices was quantified using the Folin–Ciocalteu method as described elsewhere.^4,14^ Two reagent solutions were prepared: (i) Folin–Ciocalteu reagent (1 N) diluted to a final volume of 100 mL, and (ii) sodium bicarbonate solution (6 g), added to the Folin–Ciocalteu mixture according to the referenced protocol. Samples were prepared at a concentration of 1 mg/mL in methanol. A volume of 20 μL of each sample was mixed with 150 μL of Folin–Ciocalteu reagent and allowed to stand for 5 min. Subsequently, 150 μL of sodium bicarbonate solution (NaHCO_3_) were added, and the mixture was incubated for 90 min. Absorbance was measured at 750 nm using a microplate reader (Epoch 2, BioTek). TPC was expressed as gallic acid equivalence (GAE) based on a previously constructed calibration curve.

### 2.6 Determination of Antioxidant Activity

#### 2.6.1 DPPH Free-Radical Scavenging Assay

The antioxidant capacity of the treated fruit juices was determined using the DPPH (2,2-diphenyl-1-picrylhydrazyl) assay. A 100 μM methanolic DPPH^•^ solution was prepared, and the reaction was initiated by mixing 1 mL of the sample with 1 mL of the DPPH solution.^4,7,15^ The reaction mixtures were incubated in the dark at room temperature for 30 min. Then, absorbance was measured at 515 nm using a microplate reader (Epoch 2, BioTek). The percentage of radical inhibition was calculated according to eqs 2 and 3. The antioxidant potential was expressed as Trolox equivalents based on a calibration curve.

2 $$\%\;{\mathrm{DPPH}}_{\mathrm{inhibition}}=\left[\left(\frac{A_{\mathrm{control}}-A_{\mathrm{sample}}}{A_{\mathrm{control}}}\right)\times 100\right]$$

3 $$\left[100-\left(\frac{\mathrm{Absorbance}}{\mathrm{Control}\;\mathrm{average}\;\mathrm{absorbance}}\right)\times 100\right]$$

#### 2.6.2 ABTS^•+^ Radical Scavenging Assay

The antioxidant activity of the juices was determined using the ABTS^•+^ [2,2′-azino-bis­(3-ethylbenzothiazoline-6-sulfonic acid)] radical scavenging assay.^15^ The ABTS^•+^ cation radical was generated by reacting 7 mM ABTS with 140 mM potassium persulfate (K_2_S_2_O_8_) at room temperature. The resulting solution was allowed to stand in the dark for 12–16 h, and its absorbance was adjusted to 0.70 ± 0.05 at 750 nm (*y* = −0.0003x + 0.7502, *R* ^2^ = 0.9999) using ethanol as diluent. For the assay, the ABTS^•+^ solution was mixed with the juice samples at a 1:10 (v/v) ratio in a 96-well microplate. After a 6 min reaction period, absorbance was recorded at 750 nm using a microplate reader (Epoch 2, BioTek).

#### 2.6.3 Ferric Reduction Antioxidant Power (FRAP) Assay

The Ferric Reducing Antioxidant Power (FRAP) reagent was freshly prepared by mixing 25 mL of acetate buffer (300 mmol/L), 2.5 mL of TPTZ (2,4,6-tripyridyl-s-triazine, 10 mmol/L), and 2.5 mL of FeCl_3_ (20 mmol/L). The reaction was initiated by adding 90 μL of each juice sample, 270 μL of distilled water, and 2.7 mL of the FRAP reagent into a microplate well, in triplicate.^16–18^ Absorbance was measured immediately at 595 nm using a microplate reader (Epoch 2, BioTek). The FRAP reagent was used as a blank, and ferrous sulfate (FeSO_4_) served as the calibration standard. Antioxidant activity was quantified using the FeSO_4_ calibration curve (up to 1000 μM), with results expressed as μM FeSO_4_/g of extract.

#### 2.6.4 Carotenoids Content

Carotenoids content was determined following the β-carotene-based method,^4,19,20^ Juice, distilled water, and hexane were mixed in a 1:5:6 (v/v/v) ratio, vortexed for 1 min, and centrifuged at 3000 rpm for 1 min. The resulting supernatant was collected and its absorbance measured at 450 nm using a microplate reader (Epoch 2, BioTek). Hexane served as the blank, and β-carotene was used as the calibration standard.

#### 2.6.5 UV–Vis Absorption and Fourier-Transform Infrared Spectroscopy with Attenuated Total Reflectance (FTIR-ATR) Analyses

A sample of 3 mg of each lyophilized pulp was weighed and mixed with 5 mL of ethanol. Suspensions were vortexed for 2 min and kept in ethanol for 24 h at 23 °C, protected from light. Then, the mixtures were vortexed again for 2 min and centrifuged at 3,000 rpm for 10 min. Samples were analyzed using a UV–Vis absorption spectrophotometer (Cary 50) from 200 to 800 nm. The chemical profile of the juices was determined using a FTIR–ATR spectrophotometer (Agilent Cary 630) with attenuated total reflection module (FTIR-ATR) from 680–4000 cm^–1^.

#### 2.6.6 Nuclear Magnetic Resonance (NMR) Analysis

Lyophilized pulp (50 mg) of Abricó and Cubiu was dissolved in 650 μL of DMSO-*d*_6_, stirredagitated in an ultrasonic bath for 10 min, and the resulting supernatant was transferred to a 5 mm NMR tube. NMR spectra were acquired at the Nuclear Magnetic Resonance Laboratory (NMRLab/UFAM) using a Bruker Avance III HD NMR spectrometer (Bruker, Billerica, MA, USA), operating at 11.7 T (500 MHz for ^1^H) and equipped with a 5 mm BBFO Plus SmartProbe with *Z*-axis gradient. For ^1^H NMR acquisition, the zgpr pulse sequence was used with the following parameters: 32k data points in the time domain (TD), a spectral width (SW) of 8 kHz, acquisition time (AQ) of 1.64 s, relaxation delay (D1) of 1 s, 90° pulse duration of 10 μs, receiver gain (RG) of 90.5, number of scans (NS) of 32, free induction decay (FID) resolution of 0.30 Hz, central frequency (O1) set to 1667.48 Hz, and suppression power (PLW9) of 8.6289 × 10^–5^ W. Chemical shifts (δ, in ppm) were referenced to the residual solvent peak of DMSO-*d*_6_ at δH 2.50 ppm, and coupling constants (J) were reported in Hz. Two-dimensional NMR experiments were conducted to confirm metabolite assignments, including ^1^H–^1^H correlated spectroscopy (COSY), ^1^H–^13^C heteronuclear single quantum coherence (HSQC),^21^ and ^1^H–^13^C heteronuclear multiple bond correlation (HMBC). Phase and baseline corrections of all spectra were performed manually using TopSpin 3.6.3 software (Bruker). Metabolite identification was achieved by comparing the acquired NMR data with literature values.

#### 2.6.7 Digestibility Analysis

Simulated gastrointestinal digestion was performed following the standardized INFOGEST protocol.^22,23^ All simulated fluids—simulated salivary fluid (SSF), simulated gastric fluid (SGF), and simulated intestinal fluid (SIF)—were freshly prepared prior to the assays. For the oral phase (SSF), human salivary α-amylase (Sigma-Aldrich, A1031; 75 U/mL) was dispersed in SSF and mixed with the juice samples, followed by incubation at 37 °C for 2 min. For the gastric phase (SGF), the oral bolus was diluted with SGF adjusted to pH 3.0 and supplemented with pepsin (Sigma-Aldrich, P7012; 2,000 U/mL), then incubated at 37 °C for 120 min with continuous pH monitoring and adjustment using 1 M HCl or 1 M NaOH. For the intestinal phase (SIF), the gastric chyme (20 mL) was mixed with SIF (7.8 mL), CaCl_2_·2H_2_O (40 μL; 0.3 M), ultrapure water (1.31 mL), lipase (3.2 mL; 25,000 U/mL), pancreatin (5 mL; 800 U/mL), and porcine bile extract (2.5 mL; 160 mM). The pH was adjusted to 7.0, and the mixture was incubated at 37 °C for 120 min under orbital agitation (200 rpm). Liquid aliquots were collected after each digestive phase, and nondigested solid residues were recovered by centrifugation at 10,000×*g* for 12 min. All assays were performed in triplicate.

#### 2.6.8 Statistical Analysis

A completely randomized design was adopted to evaluate the effect of ultrasound treatment at different power levels (20%, 40%, 60%, and 80%). Statistical analyses were performed for physicochemical parameters, proximate composition, mineral content, total phenolic content, and antioxidant activity (free radical scavenging assays). Analysis of variance (ANOVA) was used to assess treatment effects, and mean comparisons among the fruit juices were conducted using Duncan’s multiple range test at a significance level of *p*-value <0.05. ANOVA and Duncan tests were performed using the agricolae package, while data visualization was carried out with ggplot2. Principal component analysis (PCA) was conducted with the mixOmics package to examine correlations between antioxidant responses and ultrasound power levels. Pearson correlation analyses, implemented via the metan package, were used to evaluate linear relationships among proximate and mineral composition variables. All statistical procedures were performed in RStudio (version x64 4.2.2).

## 3 Results and Discussion

### 3.1 Proximate Composition

The physicochemical composition of the juices provides essential insight into their nutritional quality and structural integrity. Key parameters, including moisture, protein, ash, and lipid content, were quantified to characterize the proximate profile of Abricó, Cubiu, and Mari-mari juices. Figure 3 summarizes the proximate compositon for each fruit species.

**Figure 3:**
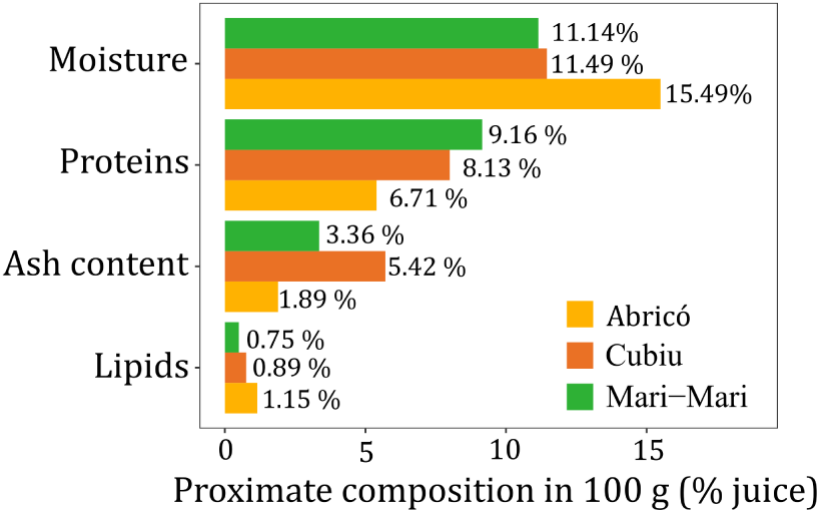
Proximate composition of Abricó, Cubiu, and Mari-mari juices (moisture, proteins, ash content, and lipids), presented as g/100 g dry weight.

The juices exhibited moisture contents of 15.49% (Abricó), 11.46% (Cubiu), and 11.14% (Mari-mari), differing by approximately 4%, which reflects their naturally succulent characteristics. The protein content was 9.16% for Mari-mari, 8.13% for Cubiu, and 6.71% for Abricó, values that exceeded those reported in the literature. Protein levels of approximately 6% was found in lyophilized juices of tucumã (*Astrocaryum aculeatum*), camu–camu (*Myrciaria dubia*), and abiu (*Pouteria caimito*),^2,23,24^ The higher protein concentrations observed in the present study indicate a promising nutritional contribution, particularly considering the role of dietary proteins in supporting metabolic health and delaying premature aging. The ash content, which reflects the mineral residue remaining after complete combustion of the sample, provides an estimate of the total mineral composition of the juices. The ash levels observed for Cubiu, Mari-mari, and Abricó (5.42%, 3.36%, and 1.89%, respectively) were higher than those reported for jambu juice (*Acmella oleracea*; 0.77–0.82%),^25^ indicating good nutritional comparability with other fruits and vegetables described in the scientific literature. Lipid contents for Cubiu, Mari-mari, and Abricó (0.89%, 0.75% and 1.15%, respectively) were also in agreement with previous findings for abiu pulp (1.28%)^26^ and araçá-boi (0.92%). These results indicated that the evaluated fruits exhibited low caloric density and are suitable for regular dietary consumption.

Table 1 presents the mineral composition (potassium, calcium, magnesium, sodium, manganese, copper, iron, and zinc) of the Abricó, Cubiu, and Mari-mari juices.

**Table 1:** Mineral Composition of Abricó, Cubiu, and Mari-Mari Juices[Table-fn tbl1fn1]

	Minerals (mg/100 g)			
Samples	K	Ca	Mg	Na
Abricó	74.4 ± 0.2^a^	23.0 ± 0.7^b^	3.4 ± 0.1^c^	2.87 ± 0.02^a^
Cubiu	45.8 ± 0.1^b^	26 ± 1^a^	15.4 ± 0.4^a^	2.03 ± 0.02^c^
Mari-mari	75 ± 3^a^	14.29 ± 0.7^c^	12.1 ± 0.4^b^	2.60 ± 0.06^b^

Minerals (mg/100 g)				
Samples	Mn	Cu	Fe	Zn
Abricó	0.40 ± 0.02c	ND	1.1 ± 0.2^b^	2.7 ± 0.3^c^
Cubiu	3.8 ± 0.1^b^	ND	1.6 ± 0.1^a^	4.3 ± 0.2^b^
Mari-mari	9.3 ± 0.2^a^	ND	1.3 ± 0.1^ab^	5.7 ± 0.2^a^

i Values are expressed as mean ± standard deviation. Means within the same column followed by different superscript letters (a–c) differ significantly according to Duncan’s test (*p*-value <0.05). K = potassium; Ca = calcium; Mg = magnesium; Na = sodium; Mn = manganese; Cu = copper; Fe = iron; Zn = zinc; ND = not detected.

The proximate composition of fruit juices is an important indicator of their nutritional quality and potential health benefits. For comparison, moisture values of 38–64% were found in pomegranate pulp,^27^ highlighting the variability among of fruit matrices, which may be influenced by intrinsic factors such as pulp-to-water ratios and tissue structure. In the present study, all analyzed minerals differed significantly among species. Potassium exhibited the highest concentrations in both sonicated and lyophilized samples, ranging from 45.8 to 75 mg/100 g. These values are comparable to those reported for other nutritionally relevant fruits, including camu–camu (44.00 mg/100 g) and duckweed pulp (750.00 mg/100 g).^28^ Overall, the mineral composition observed in Abricó, Cubiu, and Mari-mari juices suggested nutraceutical potential, reflecting favorable nutritional attributes that remained largely preserved after processing. Moreover, the incorporation of fruit flours into food products—such as breads or jams—may serve as a strategy to reduce sugar or fat content while increasing dietary fiber, thereby enhancing the functional quality of the final product.

### 3.2 pH, Titratable Acidity, and Soluble Solids

The effects of ultrasound treatment on the physicochemical properties (soluble solids, pH, and titratable acidity) of Abricó, Cubiu, and Mari-mari juices are summarized in Table 2.

**Table 2:** Physicochemical Characterization of Abricó, Cubiu, and Mari-Mari Juices Subjected to Different Ultrasound Treatments and Untreated Controls[Table-fn tbl2fn1]

Treatment (%)	No treatment	US 20 ± SD	US 40 ± SD	US 60 ± SD	US 80 ± SD
Abricó (*Mammea americana*L.)					
pH	4.46 ± 0.05^a^	4.22 ± 0.05^b^	4.16 ± 0.05^b^	4.26 ± 0.05^b^	4.16 ± 0.05^b^
TA (%)	9.73 ± 0.05^a^	7.56 ± 0.05^b^	7.03 ± 0.05^b^	7.33 ± 0.05^b^	7.36 ± 0.05^b^
SS (%)	3.36 ± 0.05^c^	2.71 ± 0.05^d^	3.46 ± 0.05^c^	3.85 ± 0.05^b^	4.11 ± 0.05^a^
Cubiu (*Solanum sessiliflorum*Dunal)					
pH	4.71 ± 0.05^a^	4.34 ± 0.05^b^	4.36 ± 0.05^b^	4.33 ± 0.05^b^	4.33 ± 0.05^b^
TA (%)	6.63 ± 0.06^a^	6.33 ± 0.05^ab^	5.91 ± 0.05^b^	4.91 ± 0.13^c^	4.81 ± 0.12^c^
SS (%)	3.33 ± 0.05^a^	2.56 ± 0.05^b^	2.43 ± 0.05^b^	2.55 ± 0.05^b^	2.43 ± 0.05^b^
Mari-mari (*Cassia leiandra*Banth)					
pH	4.13 ± 0.01^b^	4.37 ± 0.01^a^	4.47 ± 0.01^a^	4.43 ± 0.01^a^	4.40 ± 0.01^a^
TA (%)	11.50 ± 0.05^c^	11.36 ± 0.05^c^	12.33 ± 0.05^b^	12.64 ± 0.05^a^	10.40 ± 0.05^d^
SS (%)	6.83 ± 0.04^a^	6.66 ± 0.04^a^	6.71 ± 0.04^a^	6.83 ± 0.04^a^	6.63 ± 0.04^a^

i Values are expressed as mean ± standard deviation. Means in the same row followed by different superscript letters (a–d) differ significantly according to Duncan’s test (*p*-value <0.05). US = ultrasound treatment; TA = titratable acidity; SS = soluble solids.

The Abricó, Cubiu, and Mari-mari juices exhibited acidic pH values and a mildly sour sensory profile, with an average soluble solids content of 6.83 °Brix. Mari-mari juice showed the highest total acidity (12.64% TA), whereas no significant differences in pH or soluble solids were detected among treatments. These parameters remained stable after sonication and are consistent with previous reports for noni (*Morinda citrifolia* L.).^29,30^ Such stability may be attributed to the controlled cavitation effects.^26,31^ The uniformity of pH and acidity across treatments indicated that ultrasound processing did not result in physicochemical alterations capable of promoting vitamin or protein degradation.

### 3.3 Color Parameter Measurements

The colorimetric parameters of Abricó, Cubiu, and Mari-mari juices are presented in Table 3.

**Table 3:** Color Parameters (L*, a*, b*) of Abricó, Cubiu, and Mari-Mari Juices Subjected to Different Ultrasound Power Treatments[Table-fn tbl3fn1]

Treatment (%)	No treatment	US20 ± SD	US40 ± SD	US60 ± SD	US80 ± SD
Abricó (*Mammea americana*L.)					
L*	29.07 ± 0.04^e^	29.48 ± 0.02^d^	33.57 ± 0.09^c^	35.16 ± 0.03^b^	36.22 ± 0.02^a^
a*	8.11 ± 0.01^a^	7.09 ± 0.07^b^	5.9 ± 0.2^c^	5.56 ± 0.02^d^	5.93 ± 0.03^c^
b*	27.59 ± 0.05^c^	27.5 ± 0.4^c^	30.95 ± 0.02^a^	28.49 ± 0.04^b^	28.43 ± 0.05^b^
Δ*E*	—	38.4 ± 0.3^c^	40.24 ± 0.02^a^	36.81 ± 0.04^d^	36.39 ± 0.06^e^
Cubiu (*Solanum sessiliflorum*Dunal)					
L*	25.68 ± 0.05^c^	27.97 ± 0.03^a^	26.7 ± 0.4^b^	28.0 ± 0.2^a^	28.04 ± 0.04^a^
a*	7.4 ± 0.6^a^	6.29 ± 0.06^e^	6.81 ± 0.04^b^	6.68 ± 0.01^c^	6.41 ± 0.02^d^
b*	18.1 ± 0.8^e^	20.4 ± 0.7^a^	19.6 ± 0.5^b^	19.14 ± 0.05^c^	18.4 ± 0.8^d^
Δ*E*	—	32.9 ± 0.1^a^	33.1 ± 0.2^a^	31.9 ± 0.1^c^	31.1 ± 0.5^d^
Mari-mari (*Cassia leiandra*Banth)					
L*	55.01 ± 0.04^d^	58.07 ± 0.00^c^	60.14 ± 0.01^b^	61.79 ± 0.00^a^	60.15 ± 0.01^b^
a*	–4.50 ± 0.02^e^	–1.29 ± 0.01^d^	–0.61 ± 0.01^c^	–0.10 ± 0.02^b^	1.31 ± 0.02^a^
b*	21.23 ± 0.02^a^	15.77 ± 0.00^b^	15.10 ± 0.02^c^	13.44 ± 0.04^d^	13.34 ± 0.02^a^
Δ*E*	—	18.89 ± 0.01^b^	18.58 ± 0.02^c^	17.33 ± 0.04^d^	16.30 ± 0.03^e^

i Values are expressed as mean ± standard deviation. Means in the same row followed by different superscript letters (a–e) differ significantly according to Duncan’s test (*p*-value <0.05). US = ultrasound treatment; *L* = lightness; a* and b* = chromaticity coordinates; Δ*E* = total color difference.

Juice color, assessed through the CIELAB parameters L* (lightness), a* (red green), and b* (yellow-blue), is strongly influenced by pigments such as phenolics, anthocyanins, and carotenoids. Significant variations (*p*-value <0.05) were observed across treatments, characterized by increases in L* and b* values and a reduction in a*. The decrease in a* likely reflected the naturally low concentrations of red- and green-pigmenting compounds, whereas the increases in L* and b* suggested greater lightness and yellowness. This pattern is consistent with enhanced β-carotene extraction induced by ultrasonic cavitation, as previously reported for buriti juice and its byproducts.^4,32^

Ultrasound treatment caused pronounced changes in Cubiu juice, where increases in L* and b*, accompanied by a reduction in a*, indicated an intensified carotenoid release due to the ultrasound-generated shear forces,^28^ corroborating earlier findings on carotenoid quantification.^33^ A similar trend was observed for Mari-mari juice, which exhibited elevated luminosity (L* = 61.79) and reduced a* (−0.10), shifting the coloration toward greenish-yellow tones. These changes are associated with higher levels of carotenoids and vitamins A and C, which are bioactive compounds of nutritional relevance, particularly for visual and skin health.^34^ The greatest total color differences (Δ*E*) were observed in Abricó and Cubiu juices at 40% ultrasound power, primarily driven by increases in b* values linked to β-carotene enrichment, which contributes to the characteristic yellow-orange coloration of these fruits.

### 3.4 Antioxidant Activity and Total Phenolic Content (TPC) and Carotenoids Contents (CC)

Evaluating the antioxidant potential of foods has become increasingly relevant, as it provides insights into their resistance to oxidative degradation, the quantitative profile of antioxidant constituents, and their potential contribution to the body’s antioxidant defenses upon consumption.^15^ Table 4 summarizes the effects of ultrasound processing on the antioxidant activity of Abricó, Cubiu, and Mari-mari juices, as determined by DPPH^•^, ABTS^•+^, and FRAP assays.

**Table 4:** Antioxidant Activity (DPPH^•^, ABTS^•+^, and FRAP)[Table-fn tbl4fn1]

Treatment (%)	DPPH^•^	ABTS^•+^	FRAP
Abricó (*Mammea americana* L.)			
US 0	808 ± 5^l^	1171 ± 13^j^	731 ± 3^k^
US 20	879 ± 9^k^	1297 ± 5^i^	874 ± 4^i^
US 40	1029 ± 8^h^	1367 ± 10^h^	922 ± 3^h^
US 60	1125 ± 6^g^	1464 ± 12^f^	1008 ±3^g^
US 80	775 ± 6^m^	1037 ± 8^k^	702 ± 3^l^
Cubiu (*Solanum sessiliflorum*Dunal)			
US 0	582 ± 9^n^	507 ± 13^n^	686 ± 3^m^
US 20	929 ± 10^i^	316 ± 6°	607 ± 2°
US 40	900 ± 8^j^	636 ± 10^l^	629 ± 3^n^
US 60	1204 ± 7^f^	1373 ± 7^g^	1022 ± 3^f^
US 80	427 ± 10°	532 ± 11^m^	840 ± 2^j^
Mari-mari (*Cassia leiandra* Banth)			
US 0	1603 ± 9^a^	2029 ± 8^a^	1732 ± 3^a^
US 20	1549 ± 5^b^	2000 ± 20^b^	1629 ± 3^b^
US 40	1520 ± 10^c^	1929 ± 8^c^	1555 ± 4^c^
US 60	1427 ± 10^d^	1874 ± 7^d^	1477 ± 5^d^
US 80	1375 ± 9^e^	1831 ± 14^e^	1363 ± 2^e^

i Results are expressed as mean ± standard deviation (*n* = 3). Means followed by the same superscript letter within the same column do not differ significantly according to Duncan’s test (*p*-value >0.05).

Significant variations (*p*-value <0.05) in antioxidant activity were observed among the treated juices, indicating that the response to ultrasonication was dependent on both fruit matrix and ultrasound intensity. In general, moderate ultrasound intensities promoted higher radical scavenging capacity and ferric reducing power, reflecting enhanced release of antioxidant compounds induced by cavitation phenomena. These effects are associated with mechanical forces such as microjet formation and cell wall disruption, which facilitate the transfer of antioxidant molecules into the liquid phase. The observed trends are consistent with previous studies reporting improved antioxidant activity in fruit-based matrices subjected to ultrasound processing, including enhanced bioaccessibility of antioxidant compounds under moderate ultrasound conditions.^35^ The combined interpretation of DPPH^•^, ABTS^•+^, and FRAP assays provided a robust assessment of the antioxidant potential of the juices, as these methods reflected complementary mechanisms of radical scavenging and reducing capacity.

Table 5 presents the Total Phenolic Content (TPC) and carotenoid (CC) contents of Abricó, Cubiu, and Mari-mari juices subjected to different ultrasound intensities. Ultrasound processing significantly influenced the concentration of these bioactive compounds, particularly carotenoids, which are highly sensitive to structural disruption of chromoplasts and cellular membranes. In Cubiu juice, ultrasound treatment resulted in increased lightness (L*) and yellowness (b*), accompanied by a reduction in redness (a*), indicating enhanced yellow coloration. This chromatic shift is consistent with an increased release and dispersion of carotenoids, primarily β-carotene and xanthophylls, arising from cavitation-induced rupture of chromoplasts and microstreaming effects. These mechanical forces promote cell wall disruption and facilitate pigment transfer into the liquid matrix,^33^ in agreement with previous studies demonstrating improved carotenoid bioaccessibility and stability under moderate ultrasound processing.^35^ Similarly, Mari-mari juice exhibited elevated luminosity (L* = 61.79) and a slight decrease in a* (−0.10), shifting its tonality toward greenish-yellow hues. From a nutritional perspective, these color modifications reflected a higher availability of carotenoids and vitamin C bioactive compounds with synergistic antioxidant and photoprotective functions. Carotenoids serve as vitamin A precursors and contribute to visual function, epithelial maintenance, and immune regulation, whereas vitamin C is essential for collagen synthesis and protection against oxidative damage.^35,36^ The highest total color difference (Δ*E*) was detected in Abricó and Cubiu juices (≈40%), primarily driven by substantial increases in b* values associated with enhanced β-carotene accumulation. This intensified yellow–orange pigmentation not only improves the visual appeal of the juices, an important sensory factor influencing consumer acceptance, but also reflects greater nutraceutical potential, as β-carotene is a key indicator of antioxidant capacity and lipid-soluble micronutrient content.

**Table 5:** Total Phenolic and Carotenoid Contents (TPC and CC)[Table-fn tbl5fn1]

Treatment (%)	TPC	CC
Abricó (*Mammea americana* L.)		
US 0	94 ± 1^l^	18 ± 2^j^
US 20	98 ± 1^k^	34 ± 5^i^
US 40	102 ±1^j^	86 ± 2^c^
US 60	137 ± 1^h^	145 ± 54^b^
US 80	84 ± 1^m^	52 ± 3^e^
Cubiu (*Solanum sessiliflorum*Dunal)		
US 0	111 ± 2^i^	45 ± 1^f^
US 20	37 ± 1°	38 ± 20^g^
US 40	57 ± 1^n^	36 ± 3^h^
US 60	182 ± 2^f^	207 ± 1^a^
US 80	144 ± 1^g^	54 ± 36^d^
Mari-mari (*Cassia leiandra*Banth)		
US 0	838 ± 1^a^	ND
US 20	655 ± 1^b^	ND
US 40	635 ± 1^c^	ND
US 60	611 ± 1^d^	ND
US 80	607 ± 1^e^	ND

i Results are expressed as mean ± standard deviation (*n* = 3). Means followed by the same superscript letter within the same column do not differ significantly according to Duncan’s test (*p*-value >0.05). PC = phenolic content; CC = Carotenoid content; ND = not detected.

### 3.5 UV–Vis Analysis

Figure 4a presents the UV–Vis absorption spectra of Abricó juice under different conditions: untreated aqueous juice, untreated lyophilized juice extract (ethanolic solution), treated juice sonicated at 60%, and after *in vitro* gastric and intestinal digestion. All treatments exhibited absorption profiles characteristic of electronic transitions, with defined bands between 285 and 430 nm and a slight shift toward 283 nm.^37,38^

**Figure 4:**
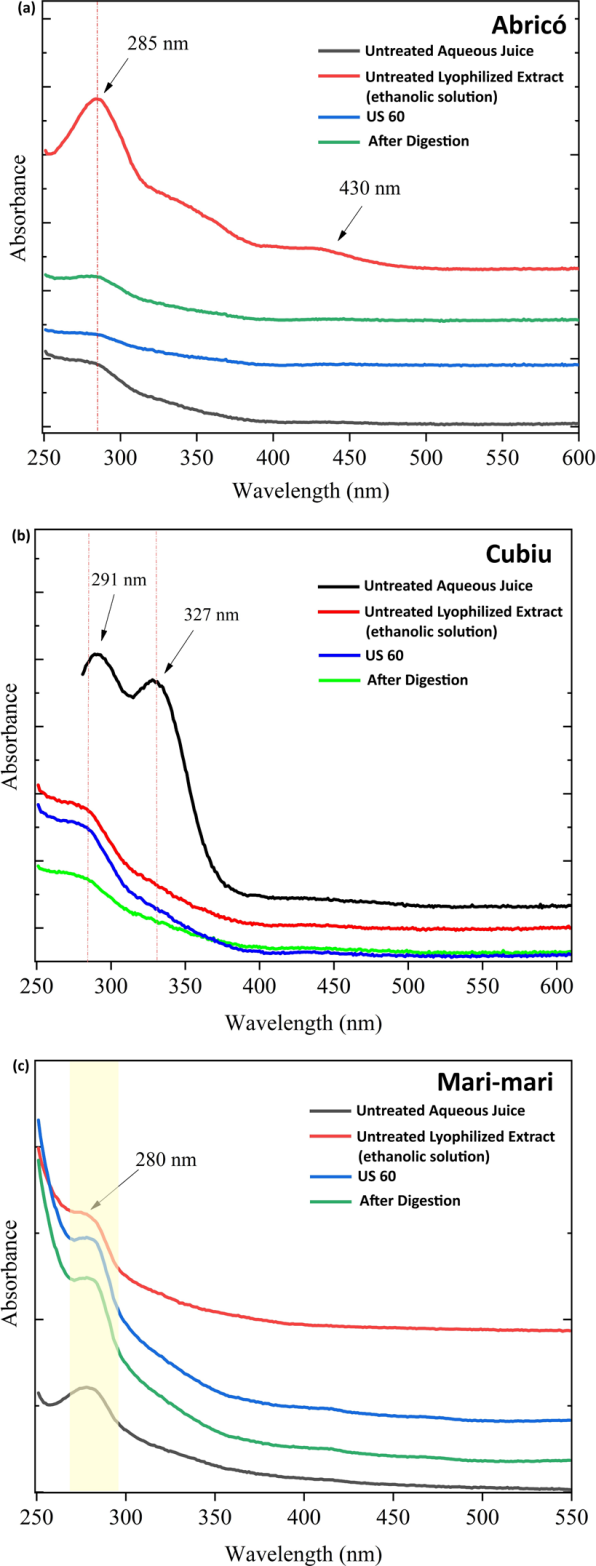
UV–vis spectra of (a) Abricó, (b) Cubiu, and (c) Mari-mari juices considering characteristic absorption bands associated with polyphenolic π → π* transitions (280–330 nm) and carotenoids (≈430 nm).

These spectral variations suggest possible modifications in the molecular environment or structural conformation of the compounds, potentially influenced by ultrasound processing. Polyphenols typically exhibit π–π* electronic transitions within 240–320 nm due to their aromatic ring systems.^39,40^ Thus, the band observed at 285 nm in Abricó juice is consistent with the excitation of π electrons into π* orbitals.

In Figure 4a (Abricó), an absorption band was also observed near 430 nm, which is characteristic of carotenoids and consistent with the 400–500 nm region typically associated with these pigments, corresponding to the S_0_ → S_2_ electronic transition.^41^ It is important to note that absorption regions may shift depending on the specific type of polyphenol or carotenoid, as well as on matrix effects or processing conditions such as ultrasound treatment and lyophilization.^42^ These electronic transitions contribute to understanding both the coloration and antioxidant behavior of the juice. In Figure 4b (Cubiu), a distinct absorption band appears near 327 nm in the untread sample, likely arising from π→π* transitions within conjugated aromatic ring systems.^41,43^

This behavior is likely associated with monomeric species linked to specific chromophores within the juice matrix, with polyphenols being the most plausible contributors. Additionally, certain carotenoids may exhibit absorption near 326–327 nm, as observed in the spectra, potentially reflecting structural modifications induced by processing.^44^

Figure 4c (Mari-mari) shows an absorption peak around 280 nm, which is typically attributed to aromatic compounds, polyphenols, flavonoids, or other chromophores containing conjugated ring systems. The observed absorption is consistent with electronic π → π* or n → π* transitions within conjugated double-bond frameworks or nonbonding orbitals.

### 3.6 FTIR Analysis

The characteristic absorption bands corresponding to the vibrational modes of functional groups present in the fruit juices were identified. Figure 5a–c displays the FTIR spectra of Abricó, Cubiu, and Mari-mari juices.

**Figure 5:**
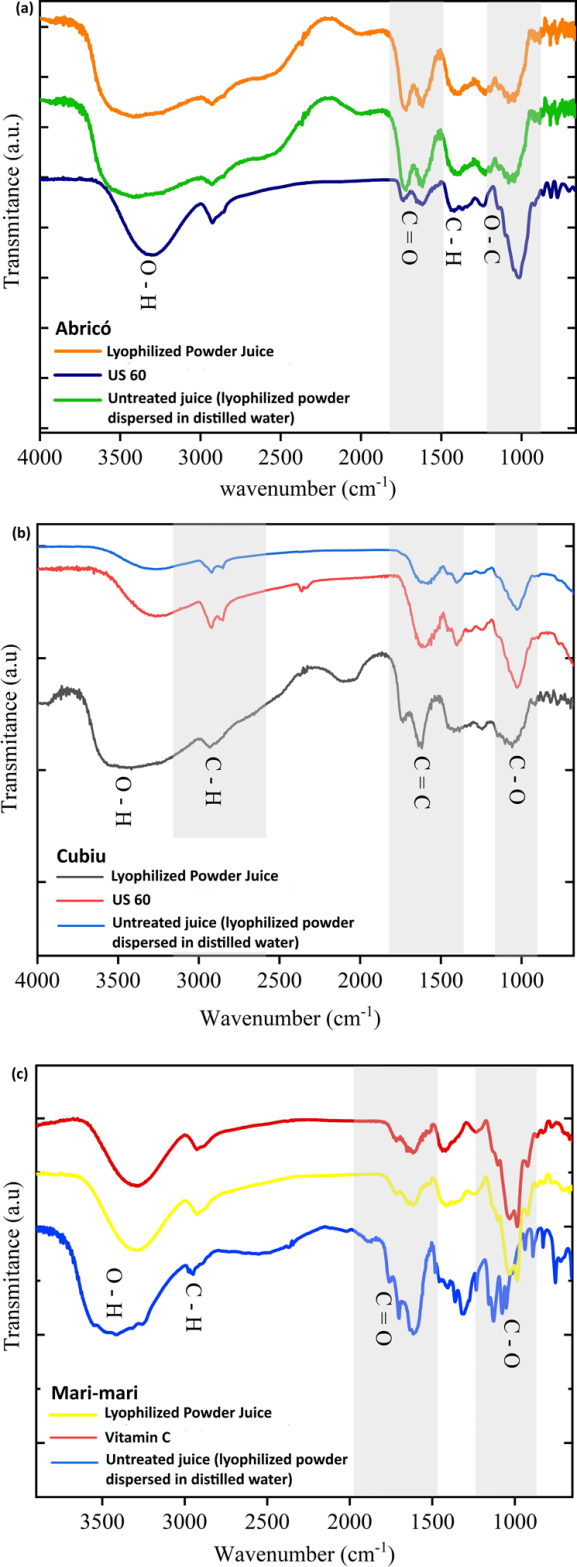
FTIR spectra of (a) Abricó, (b) Cubiu, and (c) Mari-mari juices.

Figure 5a shows the FTIR spectra of Abricó juices under three conditions: untreated juice (lyophilized powder dispersed in distilled water), untreated lyophilized power, and ultrasound-treated (60%) juice. Prominent absorption bands were observed between 2,000 and 600 cm^–1^, a region typically associated with compounds such as carotenoids and flavonoids. The main vibrational modes identified correspond to C–O, C=C, C–H, O–H, and C–O–C functional groups. A series of well-defined absorption bands also appeared within the 1,725–1,051 cm^–1^ range.^47,52^ The band at 1,725 cm^–1^ is characteristic of C=O stretching vibrations from esters or organic acids and is frequently reported in carotenoid-rich matrices. The band at 1,233 cm^–1^ is attributed to C–O stretching, while the signal around 1,153 cm^–1^ corresponds to additional C–O stretching modes. The band at 917 cm^–1^ is associated with bending (deformation) vibrations of C=C and C–O bonds.^47^ The bands detected at 1,725 and 1,233 cm^–1^ are likely linked to the presence of β-carotene (C=O stretching vibrations). Bands around 1153 cm^–1^ are assigned to C–O stretching vibrations, while those at 917 cm^–1^ are associated with the bending of C–C and C–O bonds.^50^ The absorption band at 1408 cm^–1^ is attributed to C–H bending vibrations, while those between 861 and 708 cm^–1^ correspond to out-of-plane C–H deformation modes typically associated with unsaturated systems. In the ultrasound-treated (60%) Abricó juice, the bands at 861 cm^–1^, 917 cm^–1^, and 708 cm^–1^ exhibited markedly higher intensities compared with the untreated and lyophilized juices. This enhancement may be associated to the mechanical effects of ultrasonic cavitation, which promotes structural disruption of the intracellular matrix and facilitates the release of compounds exhibiting these vibrational features. Previous studies have reported that ultrasound-assisted processing can increase the liberation of bioactive molecules in structurally dense or fibrous fruits, thereby amplifying the spectral signals associated with their functional groups.^48^

Figure 5b shows the FTIR spectra for the Cubiu juices, revealing characteristic absorption bands associated with key functional groups. The broad bands at approximately 3,476 and 3,273 cm^–1^ correspond to O–H stretching vibrations, indicative of moisture, phenolic constituents, and organic acids. The region near 2,900 cm^–1^ is dominated by aliphatic C–H stretching, commonly associated with lipids and carbohydrate structures. Absorptions at lower wavenumbers in this region may include complex contributions from the matrix but should not be directly attributed to C=C or C–O–H modes. These differences are notable between untreated and treated juices, potentially associated with matrix breakdown due to ultrasound treatment. Additionally, multiple bands in the fingerprint region may be consistent with C–O stretching vibrations. The broad band observed in the region from 1,603 to 1,681 cm^–1^ is likely due to the overlap of angular deformation of water with C=C stretching of unsaturated systems present in the matrix (such as polyenes and aromatics).^45,46^

Figure 5c presents the FTIR spectra of the Mari-mari juices, as well as a vitamin C sample, highlighting diagnostic absorption bands associated with key functional groups. The broad absorptions between 3,414 and 3,303 cm^–1^ correspond to O–H stretching vibrations, likely reflecting moisture and hydroxyl-rich compounds present in the matrix. The bands at 2,946 and 2,927 cm^–1^ were assigned to aliphatic C–H stretching modes, including symmetric and asymmetric vibrations of methyl and methylene groups.^35^ Similarly, the band at 1,429 cm^–1^ is attributed to deformation (bending) modes of these same functional groups. A low-intensity feature near 1,896 cm^–1^, consistent with previous reports, may arise from overtone or combination bands commonly observed in complex aromatic systems.^46,53^ The absorption region between 1,726 and 1,759 cm^–1^ corresponds to C=O stretching vibrations of carbonyl groups, characteristic of flavonoid structures. These spectral signatures collectively support the presence of phenolic, aromatic, and carbohydrate-related compounds.

### 3.7 Principal Component Analysis (PCA)

Figure 6 presents the PCA score and loading plots for Abricó, Cubiu, and Mari-mari juices subjected to different ultrasound intensities. The first two principal components explained 87% of the total variance, with PC1 accounting for 76% and PC2 for 11%, indicating a robust representation of the data set.

**Figure 6:**
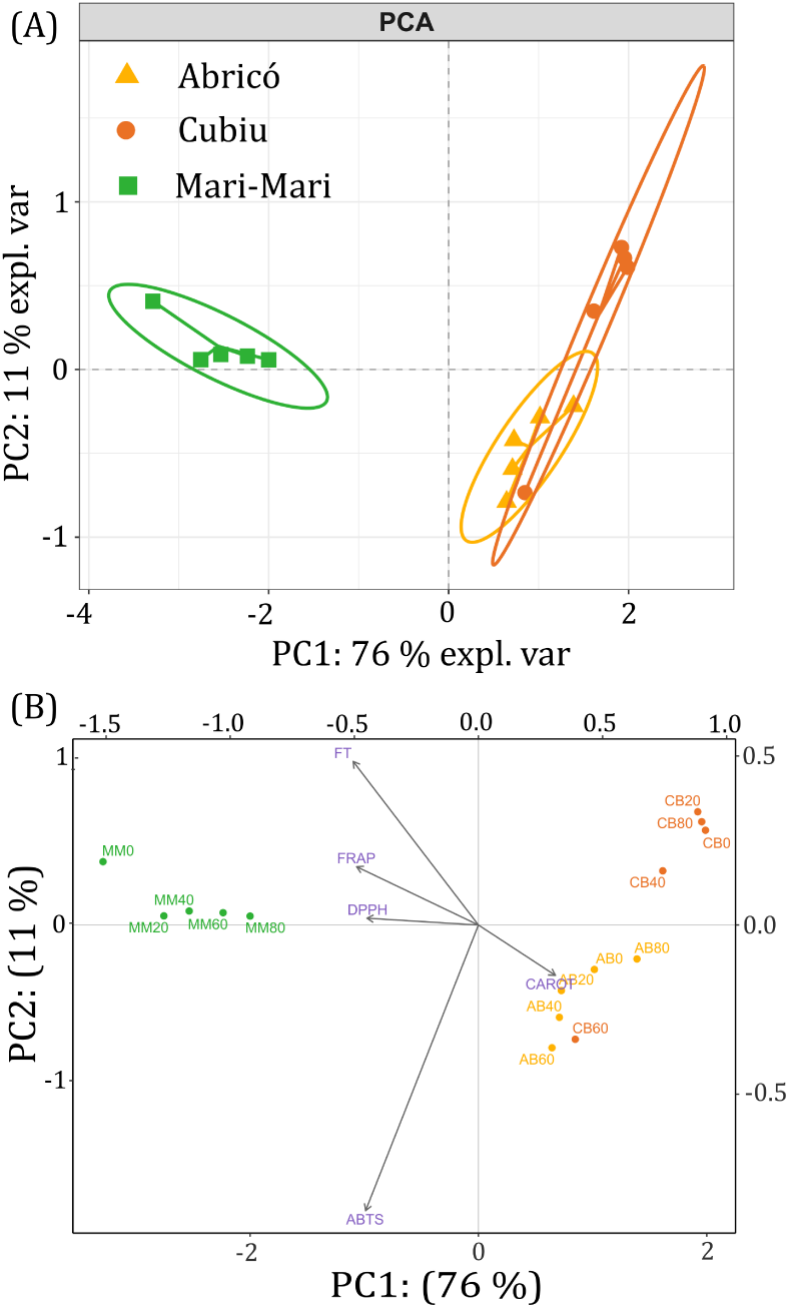
Principal component analysis (PCA) of Abricó, Cubiu, and Mari-mari juices subjected to different ultrasound treatments. (a) Score plot showing sample distribution along PC1 (76% of explained variance) and PC2 (11% of explained variance), with clear clustering by fruit species. (b) Loading plot illustrating the contribution of antioxidant parameters (DPPH, ABTS, FRAP), phenolic compounds (FT), and carotenoids (CAR) to sample separation. Abricó, Cubiu, and Mari-mari samples are represented by triangles, circles, and squares, respectively, with ellipses highlighting group clustering patterns.

The score plot (Figure 6a) revealed a clear clustering pattern primarily driven by fruit species, reflecting intrinsic differences in matrix composition. Abricó and Cubiu samples clustered closely along PC1 due to their compositional similarity, mainly associated with higher contents of dietary fibers and carotenoids, whereas Mari-mari samples formed a distinct group characterized by higher concentrations of vitamins, and specific bioactive compounds. Within each fruit group, variations in ultrasound intensity influenced the dispersion of samples along the principal components, indicating a matrix-dependent response to ultrasound processing. These findings are related to previous report,^5^ revealing strong Pearson correlations between phenolic content and antioxidant activity in açaí and buriti juices, further supporting the observed clustering pattern.

The loading plot (Figure 6b) indicates that carotenoids presented high loadings on PC1 and are closely associated with Abricó and Cubiu samples, whereas PC2 is primarily driven by antioxidant assays (DPPH, ABTS, FRAP) and total phenolics, explaining the separation of Mari-mari samples. The distribution patterns along both principal components, evidenced by their respective positive and negative score values, established significant correlations with antioxidant assays.

This systematic variation confirmed the substantial antioxidant capacity of these juices, primarily attributable to their phenolic compounds and vitamins A and C content. These findings are aligned with previous work^54^ reporting polyphenols as major contributors to antioxidant activity in spice matrices.

### 3.8 Correlation

Figure 7 shows the Pearson correlation matrix between proximate composition parameters and mineral content in Abricó, Cubiu, and Mari-mari juices. Strong positive correlations were observed between ash content and iron (*r* = 0.945) and magnesium (*r* = 0.860), indicating that ash content is a reliable indicator of total mineral contribution in these juices. Protein content exhibited an exceptionally strong correlation with zinc (*r* = 0.984), which was also strongly correlated with manganese (*r* = 0.953), suggesting coordinated accumulation of these micronutrients within the fruit matrix. These patterns are consistent with previous report^52^ for jambu (*Acmella oleracea*), where proximate composition was closely linked to mineral distribution.

**Figure 7:**
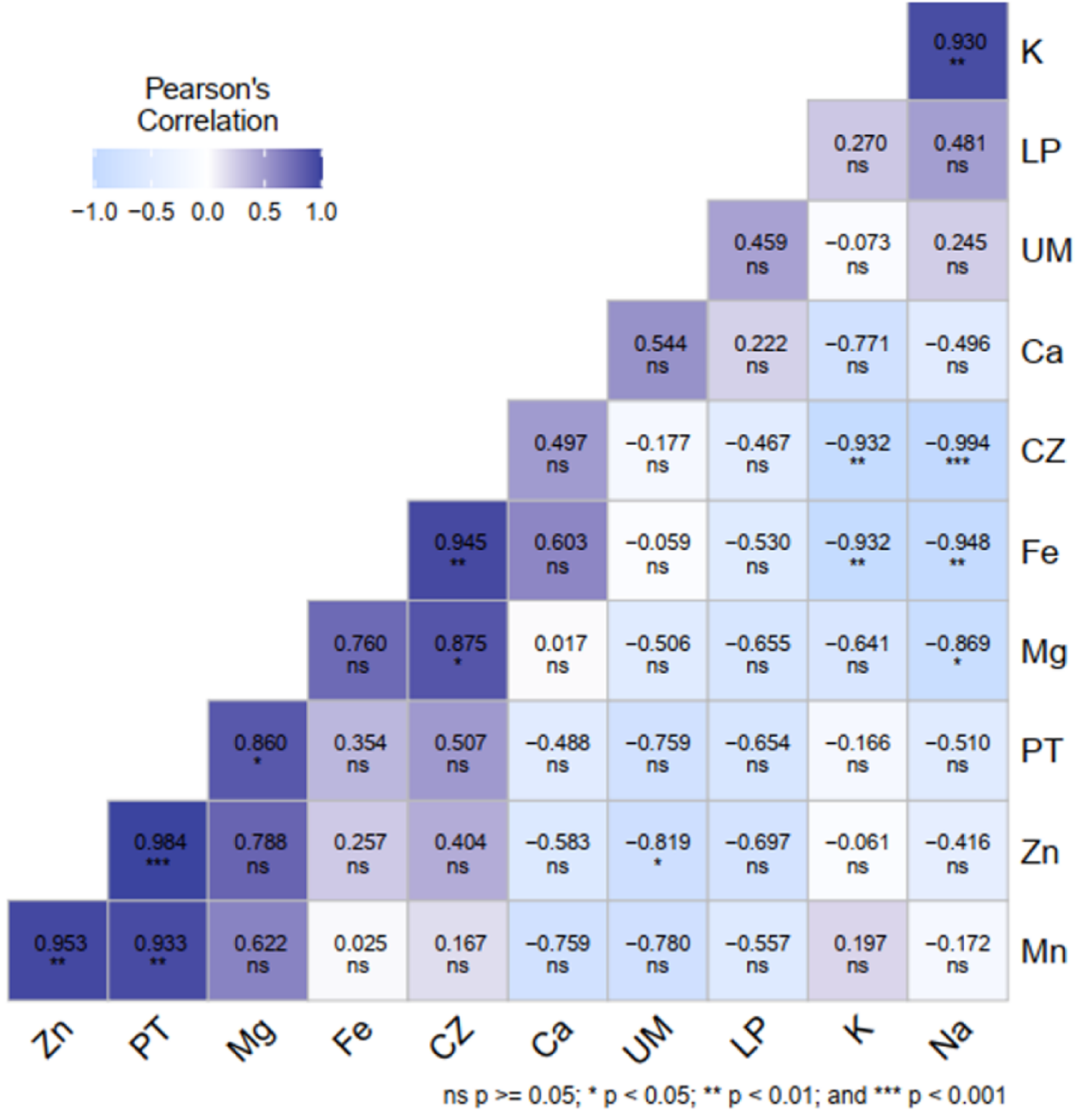
Pearson correlation matrix showing the relationships between proximate composition variables (PT = proteins; CZ = ash; UM = moisture; LP = lipids) and mineral elements (Zn = zinc; Ca = calcium; Fe = iron; Mg = magnesium; Mn = manganese; Na = sodium; K = potassium) in Abricó, Cubiu, and Mari-mari juices. Statistical significance is indicated as *p < 0.05, **p < 0.01, and ***p < 0.001. Cells without asterisks do not differ significantly according to Duncan’s test (*p*-value >0.05).

Negative correlations were observed between lipid content and several minerals, including iron, magnesium, zinc, and manganese, indicating a limited role of lipids in mineral retention in the aqueous juice matrix. Additionally, ash content showed a strong negative correlation with potassium and sodium (*r* = −0.932), which may reflect dilution effects or the presence of nonmineral ash components.^55^ Protein content was negatively correlated with calcium, potassium, and sodium, reinforcing compositional differences among the studied fruits.

These mineral/nutrient interactions are consistent with previous reports on plant-based matrices and highlight the nutritional relevance of these juices as sources of essential minerals, particularly iron, magnesium, zinc, and manganese.^54,56^ These observations are consistent with previous reports reporting similar mineral-nutrient interactions.^7,11^

### 3.9 NMR Analysis and Compound Variability

The DMSO-*d*_6_ extracts of Abricó and Cubiu were analyzed by ^1^H NMR spectroscopy combined with two-dimensional experiments (COSY, HSQC, and HMBC), allowing the identification of the main metabolites Table 6, Figure 8). The spectra displayed signals in three distinct regions: aliphatic (δ 0.50–2.30), carbinolic (δ 2.31–6.00), and aromatic (δ 6.01–8.80) compounds. Metabolite assignments were confirmed by comparing chemical shifts, coupling constants, and ^1^H–^13^C correlations with literature data and validated databases on HMDB (Human Metabolome Database) and BMRB (Biological Magnetic Resonance Bank).

**Table 6:** Metabolites Identified by ^1^H NMR Spectroscopy in Abricó and Cubiu Extracts along with Their Respective Spectral Information^a^

No.	Compound	δH (ppm, multiplicity, *J* in Hz)	δC (ppm)	HMBC correlations	Species
1	Fatty acids	0.85 (*t*, 7.0); 0.87 (*t*, 6.0); 1.05 (*t*, 7.0)	14.4; 18.1; 14.7	–	Both
2	Acetic acid	1.81 (*s*)^a^; 1.91 (*s*)^b^	28.3	177.2	Both
3	Quinic acid	1.61 (*dd*, 13.5, 7.0); 1.81 (*dd*, 17.0, 4.0)	39.6; 44.5	70.5; 76.3; 73.3	*S. sessiliflorum*
4	Malic acid	2.37 (*dd*, 15.7, 5.0); 2.65 (*dd*, 12.0, 4.3)	41.3	67.0; 173.9	*S. sessiliflorum*
5	Citric acid	2.51 (*d*, 15.2); 2.68 (*d*, 15.1)	171.9 (C=O)	48.4; 77.9	*S. sessiliflorum*
6	α, β-Glucose	3.17–5.23 (*m*)	61.2–97.0	73.8; 72.4	Both
7	Sucrose	3.50–5.40 (*m*)	60.5–104.8	74.1; 82.2; 75.0	Both
8	Syringic acid	7.37 (*s*)	112.1 (C-2/C-6); 172.3 (C=O)	153.3; 62.4	*M. americana*
9	Gallic acid	7.24 (*s*, H-2/H-6)	115.6	151.2; 144.1	*M. americana*
10	Chlorogenic acid	7.05 (*d*, 2.0); 7.42 (*d*, 15.9); 6.17 (*d*, 15.9)	114.3; 148.8; 145.1	115.4; 122; 168.8	*S. sessiliflorum*
11	*p*-Coumaric acid	7.42 (*d*, 15.9); 6.17 (*d*, 15.9); 6.98 (*dd*, 8.0, 2.0)	145.1; 114.6; 121.2	130.9; 125.4; 167.7	*S. sessiliflorum*

a Nd—Not identified.

**Figure 8:**
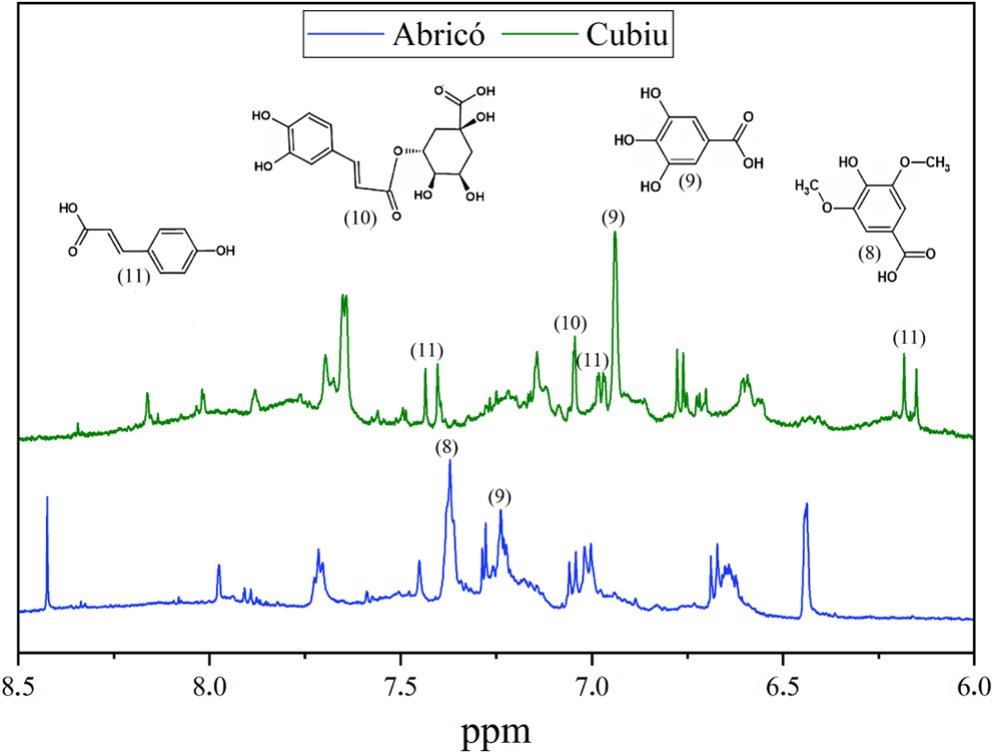
^1^H NMR spectra of aqueous extract of Abricó and Cubiu juices treated with probe ultrasound and in vitro digestibility.

The ^1^H NMR spectra for Abricó and Cubiu are shown in Figure 8. The spectrum displayed a complex pattern, with signals attributed to aliphatic (d 0.50–2.30), carbinolic (d 2.31–6.00) and aromatic (d 6.01–8.80) compounds. The identified metabolites were compared with chemical shift data, coupling constants, and correlations obtained from two-dimensional experiments, referencing the literature and scientific databases such as HMDB and BMRB. Additionally, 2D NMR experiments were conducted, including correlated spectroscopy (^1^H–^1^H) COSY, (^1^H–^13^C) HSQC, and (^1^H–^13^C) HMBC.

In the aliphatic region, long-chain fatty acids (1) were identified by terminal methyl signals at δH 0.85–1.05 (*t*), with HSQC correlations to δC 14.4–18.1. Acetic acid (2) showed characteristic singlets at δH 1.81 (Abricó) and δH 1.91 (Cubiu). Quinic acid (3), exclusive to Cubiu, displayed signals at δH 1.61 and 1.81 (*dd*), correlating with δC 39.6 and 44.5. The carbinolic region was richer in Cubiu extracts, revealing important organic acids and carbohydrates. Malic acid (4) showed diastereotopic hydrogens at δH 2.37 and 2.65 (*dd*, δC 41.3), while citric acid (5), also exclusive to Cubiu, exhibited signals at δH 2.51 and 2.68 (*d*) with HMBC correlation to the carbonyl at δC 171.9. α,β-Glucose, which has already been identified in the leaves of this species,^57^ (6) and sucrose (7) were present in both species, with significantly higher intensities in Cubiu. The aromatic region showed greater abundance in Abricó, indicating higher phenolic content. Syringic acid (8) displayed a singlet at δH 7.37 (δC 112.1, C-2/C-6) with HMBC correlation to the carbonyl (δC 172.3). Gallic acid (9) showed δH 7.24 (*s*, H-2/H-6, δC 115.6). In Cubiu, chlorogenic acid (10) was characterized by signals at δH 7.05 and 7.42 (*d*) correlating with δC 114.3 and 148.8, while *p*-coumaric acid (11) showed diagnostic signals at δH 7.42, 6.17, and 6.98 (δC 145.1, 114.6, 121.2).

These assignments are consistent with previous phytochemical studies of *Solanaceae* and *Clusiaceae* species and corroborated the FTIR analysis, where absorption bands at 1,743 cm^–1^ (carboxylic acids), 1,604/1,514 cm^–1^ (aromatics), and 2,924/2,854 cm^–1^ (aliphatic chains) confirmed the presence of the identified compound classes. The complementary use of FTIR (functional groups) and 2D NMR (structural elucidation) provided robust evidences for the metabolite assignments in Table 6.

### 3.10 Bioaccessibility Analysis

During gastrointestinal digestion, variations in pH and enzymatic activity can reduce the concentration of native bioactive compounds. Accordingly, the bioaccessibility of the major bioactives in Abricó and Cubiu juices—primarily carotenoids—relative to their phenolic content is presented in Figure 9.

**Figure 9:**
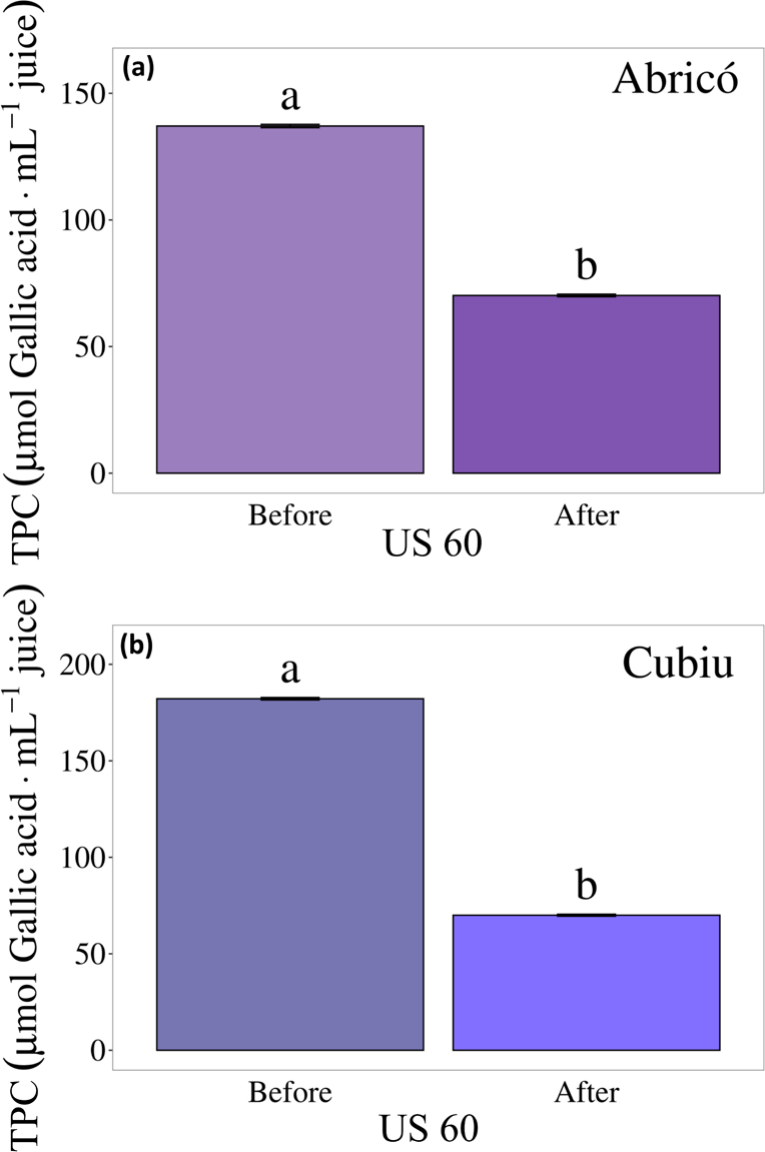
(a,b): Total Phenolic Compound (TPC) content during simulated *in vitro* digestibility of Abricó and Cubiu.

Abricó juice (Figure 9a) exhibited a markedly higher total phenolic content (TPC) prior to *in vitro* digestion, with a significant reduction observed following the simulated gastrointestinal process, indicating substantial degradation or transformation of phenolics during digestion. A similar pattern was observed for Cubiu juice (Figure 9b), which also presented high initial TPC followed by a statistically significant decline after digestion. These results suggested that a considerable fraction of the phenolic compounds present in both juices is not bioavailable under the simulated gastrointestinal conditions.

The reduction in TPC observed after *in vitro* digestion is likely related to the partial release of phenolic compounds from the food matrix, followed by their degradation under gastric conditions. The gastric phase, characterized by highly acidic pH values (1.5–3.0), can promote the hydrolysis of glycosidic bonds in flavonoids and accelerate the degradation of anthocyanins, which are particularly unstable in acidic environments. A significant decrease (*p*-value <0.05) in TPC was detected in the 60% sonicated juices of Abricó (139.65 ± 0.64 to 78.21 ± 1.55 mg GAE/mL) and Cubiu (182.32 ± 0.87 to 78.59 ± 1.63 mg GAE/mL). These findings indicated that, before digestion, both juices contained high concentrations of phenolics and carotenoids,^50^ supporting their strong antioxidant profiles. However, during the intestinal phase, substantial losses occurred, with approximately 56% of phenolics remaining in Abricó and 43% in Cubiu juices, suggesting that only a fraction of these compounds becomes bioavailable for absorption.^58^ These results, presented in Figure 10, highlighted the dietary importance of compounds and their potential contributions to health.

**Figure 10:**
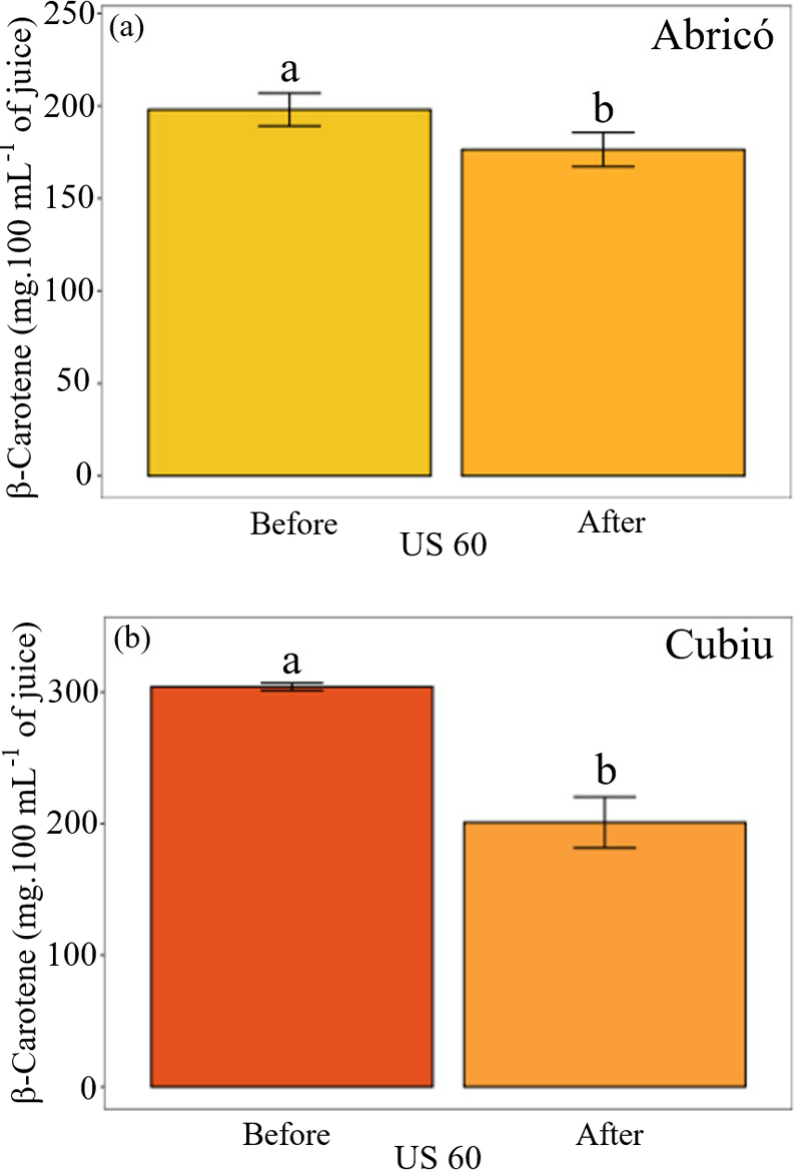
Carotenoid content in (a) Abricó and (b) Cubiu juices before (undigested) and after *in vitro* digestion (simulated gastric and intestinal phases; oral phase excluded). Values are expressed as mean ± standard deviation (*n* = 3). Carotenoids were quantified as key bioactive constituents in juices treated with 60% ultrasound power. Different lowercase letters (a, b) within each fruit indicate statistically significant differences between digestion stages (*p*-value <0.05).

Previous research,^4^ explored the bioavailability of bioactive compounds released through ultrasound in buriti and pear juices, both before and after digestion, corroborating the findings of this study. Additionally, studies have focused on the phenolic composition of *Solanum sessiliflorum* and *Eugenia stipitate*,^59,60^ demonstrating promising results in the presence of various phenolic compounds beneficial for health and potential in preventing oxidative stress-related diseases. These investigations underscore the significance of phenolic content in fruits overall and particularly in apricot and cubiu (Figure 10a), known for their richness in diverse bioactive compounds.^32,61^ However, carotenes, β-carotene, and flavonoids are being considered as agents in combating premature aging, diseases induced by oxidative stress, and promoting health and disease prevention.^26^ Intriguingly, the release of compounds within the intracellular wall of vegetables and fruits is more efficient with the application of ultrasound technique.

In the digestibility assay, the carotenoid concentration in the predigestion and gastric phases remained stable at approximately 76.42 ± 4.44 mg·100 mL^–1^, showing a significant difference (*p*-value <0.05) between the pregastric and postdigestion phases. The observed stability of carotenoids in Abricó juice (Figure 10a) is likely associated with the protective effect of its lipophilic matrix, which facilitates micelle formation and reduces oxidative degradation during digestion.^4,32^ In contrast, (Figure 10b) Cubiu juice exhibited a significant reduction in β-carotene content after digestion, indicating lower bioaccessibility. This decline may be attributed to oxidative and enzymatic degradation of carotenoids under acidic and oxidative gastrointestinal conditions, consistent with the known sensitivity of β-carotene to light, temperature, and pH.^2,62^ Similar patterns have been reported in studies of fresh tucumã pulp from Amazonas and Pará (Brazil), where the concentrations of tocopherols and vitamins A and E were comparable to the values observed here.^63,64^

Overall, the changes observed before and after the *in vitro* digestion highlighted the dynamic nature of carotenoid bioaccessibility in these Amazonian fruits. These findings emphasize the relevance of understanding how digestion influences the release and absorption of key bioactive compounds, with direct implications for their nutritional and health-promoting potential.

### 3.11 Industrial Relevance, Limitations, and Future Perspectives

From an industrial perspective, the results of the present study demonstrated that water-based ultrasound-assisted extraction operated at moderate intensities represents a promising and sustainable strategy for enhancing the release and bioaccessibility of antioxidant compounds in Amazonian fruit juices. The use of water as a green solvent, combined with reduced processing time and energy input, supports the potential scalability of this approach for the production of functional beverages and nutraceutical ingredients. Moreover, the matrix-dependent response observed among Abricó, Cubiu, and Mari-mari highlights the importance of tailoring ultrasound parameters to specific botanical characteristics. Despite these advantages, some limitations should be acknowledged. The scalability of ultrasound-assisted extraction remains a key challenge, as cavitation efficiency and energy distribution in laboratory-scale systems may differ from pilot- or industrial-scale operations. In addition, the intrinsic variability of Amazonian fruits associated with seasonality, ripening stage, and geographical origin may affect the reproducibility and standardization of bioactive compound recovery. Future research may focus on optimizing operational parameters to accommodate raw material variability, as well as exploring the integration of ultrasound with other emerging technologies.

## 4 Conclusions

The present study provides a comprehensive physicochemical, structural, and spectroscopic evaluation of Abricó (*Mammea americana*L.), Cubiu (*Solanum sessiliflorum* Dunal), and Mari-mari (*Cassia leiandra* Banth) juices, confirming their remarkable nutritional and functional potential. Their composition—characterized by high levels of vitamins, minerals, and dietary fiber, combined with low lipid content and high moisture—supports their classification as nutrient-dense, low-calorie matrices suitable for functional food applications.

Ultrasound-assisted processing (20–80%) proved to be an effective nonthermal technology for enhancing the extraction of bioactive compounds while preserving the physicochemical stability of the juices. Moderate ultrasound intensities, particularly 60%, resulted in increased carotenoid and phenolic contents, leading to greater antioxidant capacity, as demonstrated by the DPPH^•^, ABTS^•+^, and FRAP assays. Improvements in color parameters, such as increased lightness (L*) and yellowness (b*), were associated with enhanced β-carotene extraction, especially in Abricó and Cubiu juices.

Spectroscopic analyses (UV–Vis and FTIR) revealed molecular fingerprints characteristic of polyphenols, carotenoids, and flavonoids, indicating structural preservation and potentially improved bioavailability of antioxidant compounds. Multivariate (PCA) and correlation analyses further demonstrated consistent positive associations among phenolics, carotenoids, minerals, and antioxidant activity, with Mari-mari forming a distinct cluster due to its higher vitamin contents.

Overall, ultrasound emerges as a sustainable and eco-efficient processing strategy for improving the functional and nutraceutical quality of Amazonian fruit juices without compromising their compositional integrity. These findings provided mechanistic insights into the structural and compositional transformations induced by sonication and highlight the potential of Abricó, Cubiu, and Mari-mari as promising natural sources of antioxidants for the development of high-value functional beverages aligned with global trends toward health-promoting and sustainable foods.

## Data Availability

The data used to support the findings of this study are available from the corresponding author upon request.
